# Prolonged Sitting Interrupted by 6-Min of High-Intensity Exercise: Circulatory, Metabolic, Hormonal, Thermal, Cognitive, and Perceptual Responses

**DOI:** 10.3389/fphys.2018.01279

**Published:** 2018-10-16

**Authors:** Billy Sperlich, Ine De Clerck, Christoph Zinner, Hans-Christer Holmberg, Birgit Wallmann-Sperlich

**Affiliations:** ^1^Integrative and Experimental Exercise Science & Training, Institute of Sport Science, University of Würzburg, Würzburg, Germany; ^2^Artevelde University College Ghent, Ghent, Belgium; ^3^Department of Sport, University of Applied Sciences for Police and Administration of Hesse, Wiesbaden, Germany; ^4^School of Sport Sciences, UiT The Arctic University of Norway, Tromsø, Norway; ^5^Department of Physiology and Pharmacology, Karolinska Institute, Stockholm, Sweden; ^6^Institute of Sport Science, University of Würzburg, Würzburg, Germany

**Keywords:** inactivity, high-intensity interval training, sedentary lifestyle, students, workplace, intervention, physical activity, health promotion

## Abstract

The aim was to examine certain aspects of circulatory, metabolic, hormonal, thermoregulatory, cognitive, and perceptual responses while sitting following a brief session of high-intensity interval exercise. Twelve students (five men; age, 22 ± 2 years) performed two trials involving either simply sitting for 180 min (SIT) or sitting for this same period with a 6-min session of high-intensity exercise after 60 min (SIT+HIIT). At T_0_ (after 30 min of resting), T_1_ (after a 20-min breakfast), T_2_ (after sitting for 1 h), T_3_ (immediately after the HIIT), T_4_, T_5_, T_6_, and T_7_ (30, 60, 90, and 120 min after the HIIT), circulatory, metabolic, hormonal, thermoregulatory, cognitive, and perceptual responses were assessed. The blood lactate concentration (at T_3_–T_5_), heart rate (at T_3_–T_6_), oxygen uptake (at T_3_–T_7_), respiratory exchange ratio, and sensations of heat (T_3_–T_5_), sweating (T_3_, T_4_) and odor (T_3_), as well as perception of vigor (T_3_–T_6_), were higher and the respiratory exchange ratio (T_4_–T_7_) and mean body and skin temperatures (T_3_) lower in the SIT+HIIT than the SIT trial. Levels of blood glucose and salivary cortisol, cerebral oxygenation, and feelings of anxiety/depression, fatigue or hostility, as well as the variables of cognitive function assessed by the Stroop test did not differ between SIT and SIT+HIIT. In conclusion, interruption of prolonged sitting with a 6-min session of HIIT induced more pronounced circulatory and metabolic responses and improved certain aspects of perception, without affecting selected hormonal, thermoregulatory or cognitive functions.

## Introduction

There is convincing evidence that physical activity (PA) prevents and treats a wide range of physical and psychological disorders, as well as promoting longevity (Physical Activity Guidelines Advisory Committee, 2008). The WHO recommendations for health-related PA for adults are at least 150 min of moderate-intensity or 75 min of vigorous-intensity aerobic PA or an equivalent combination of both each week (World Health Organization, 2010). However, the common assumption that sufficient moderate-to-vigorous PA compensates for sedentary behavior is refuted by current findings that prolonged sitting time is associated with increased all-cause mortality (Chau et al., 2013) and numerous other negative health conditions, such as obesity (Healy et al., 2008), cardiovascular diseases (Dunstan et al., 2011; de Rezende et al., 2014), and type 2 diabetes mellitus (Wilmot et al., 2012), as well as various other metabolic risk factors (Sisson et al., 2009) independent of PA levels. However, a harmonized meta-analysis of data from more than one million men and women showed that high levels of moderate-to-vigorous PA (i.e., about 60–75 min per day) seemed to eliminate the increased risk of death associated with high sitting time and provided further evidence for the benefits of moderate-to-vigorous PA in societies where people have to sit for long periods during work (Ekelund et al., 2016).

In developed countries most adults sit for long periods and occupational sitting is a primary contributor to daily sedentary time in white-collar workers (Parry and Straker, 2013; Wallmann-Sperlich et al., 2014), who spend more than 70% of their working hours sitting (Thorp et al., 2012; Wallmann-Sperlich et al., 2017). Recent findings suggest that prolonged sitting in the workplace has deleterious health consequences (van Uffelen et al., 2010) and initial intervention studies indicate that interrupting such prolonged sitting reduces these risks for physically inactive and/or overweight or obese individuals (Dunstan et al., 2012; Howard et al., 2013). However, such short-term improvement in the metabolic profile could not be observed in physically active participants (Altenburg et al., 2013). In contrast, in their review Benatti and Ried-Larsen (2015) conclude that breaking up prolonged sitting time with exercise of higher intensity or longer duration more effectively promotes positive outcomes in young individuals who are physically active (Benatti and Ried-Larsen, 2015). From the employer’s point of view, prolonged breaks for exercise during work are problematic, since lack of time is a main barrier to more physical activity (Trost et al., 2002), especially in the workplace setting. Hence, PA breaks need to be as time-efficient as possible and to provide the best possible health effects.

Strategies designed to improve cardiovascular and metabolic health in individuals with a variety of diseases have begun to replace low-intensity exercise with repeated short or longer bouts of relatively high-intensity interval exercise (HIIT) (Gibala et al., 2012; Kessler et al., 2012; Elliott et al., 2014; Little and Francois, 2014; Gielen et al., 2015; Messler et al., 2016; Schmitt et al., 2016).

High-intensity interval exercise requires rapid and repeated muscle contractions involving aerobic and anaerobic energy production, inducing pronounced circulatory, metabolic and hormonal responses (e.g., elevated heart rate, blood flow, and levels of stress hormones), and resulting in significant heat production. This heat production stimulates in turn responses that dissipate heat, including enhanced subcutaneous blood flow, sweat production and, finally, overall body temperature, thereby causing heat stress that further elevates levels of stress hormones (Brenner et al., 1998) and may impair well-being (Gleeson, 1998; Gavin, 2003; Skurvydas et al., 2008). Moreover, business apparel such as a suit or long-sleeve shirt hamper heat loss and evaporation of sweat from the skin, thereby promoting sensations of wetness, shivering and sweating (Aoyagi et al., 1998; Gavin, 2003; Dai et al., 2008) and potentially distracting the worker from tasks that require concentration.

Previous reports suggest that exercise of low-to-moderate intensity does not influence executive functioning, but that when the physical workload becomes heavy, exercise impairs cognitive functions (Executive and non-Executive) (Mekari et al., 2015). Studies on cerebral oxygenation indicate that lower availability of oxygen slows down reaction time only in connection with executive tasks (Mekari et al., 2015). Perceived fatigue is associated with fatigability during HIIT, with lowered saturation of hemoglobin in the cerebral cortex with oxygen during sprints (Monroe et al., 2016). Neither cognitive function nor cerebral oxygenation have yet been investigated in connection with performance of HIIT at work.

Since HIIT is an attractive time-efficient strategy for improving certain aspects of health, the question arises as to whether, when applied in the workplace this type of physically and mentally strenuous exercise with considerable heat production distracts the worker from tasks that require concentration. The present study was designed to examine certain aspects of circulatory, metabolic, hormonal, thermoregulatory, cognitive, and perceptual responses upon sitting after a brief bout of high-intensity exercise.

## Materials and Methods

### Study Participants and Protocol

The twelve students (five men and seven women; age, 22 ± 2 years; height, 174 ± 7 cm; body mass, 66.2 ± 9.9 kg, **Table 1**) who volunteered were right-handed and preparing for their final exams of the semester.

**Table 1:** Anthropometric and physiological characteristics of the study participants (*n* = 12).

Characteristics	Means ± SD (95% CI)
Age [years]	22 ± 2 (20–23)
Body mass [kg]	66.2 ± 9.9 (59.9–72.5)
Height [cm]	174 ± 7 (170–179)
Body mass index [kg⋅m^-2^]	21.7 ± 2.1 (20.4–23.1)
Body fat [%]	22.5 ± 4.7 (19.6–25.5)
Peak heart rate [beats⋅min^-1^]	196 ± 4 (193–198)
Peak oxygen uptake [ml⋅min^-1^]	3230 ± 942 (2630–3830)
Peak oxygen uptake [ml⋅min^-1^⋅kg^-1^]	48.2 ± 9.0 (42.4–53.9)
Caloric intake during breakfast [kcal⋅kg^-1^]	11.0 ± 5.4 (7.6–14.4)


**FIGURE 1:**
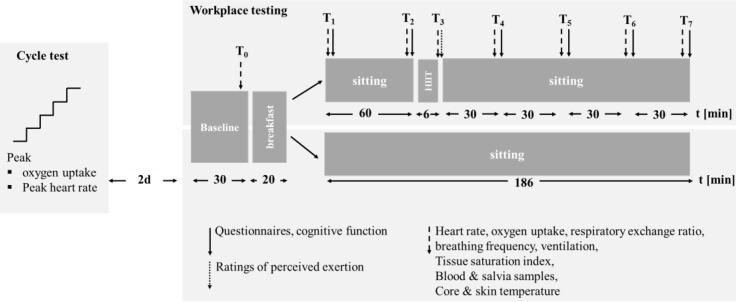
The overall design of the present study.

None reported any health disorders, and all provided their written informed consent to participate prior to initiation of the study. The major inclusion criteria were (1) enrollment as a student preparing for the final exams of the semester and (2) no routine experience of HIIT. All procedures were conducted in accordance with the Declaration of Helsinki and the experimental protocol was approved by the ethical review board of the Sport Science Institute of the University of Würzburg. The study protocol is illustrated in **Figure 1**.

All participants were asked to report well-hydrated and to refrain from consuming alcohol and caffeine for at least 24 h, as well as from engaging in strenuous exercise for at least 48 h prior to all testing. In connection with the first visit, body and fat-free mass were determined with a four-electrode impedance scale and peak values of heart rate and oxygen uptake assessed during a ramp-like cycle (Cyclus II, Avantronics, Leipzig, Germany) test (70 W; incremental increase of 30 W every min) until volitional exhaustion.

Then, 48 h later all participants reported in a fasted state to an office workplace with climate control and adjustable desks and chairs. After calibration of the gas-exchange apparatus and application of the facial mask and chest belt for recording heart rate, each participant rested lying down for 30 min and thereafter ate a 20-min breakfast of toast with jelly and a cup of green tea without sugar. Next, the electrodes of the near-infrared spectroscope were attached to the participant’s forehead, after which he/she sat for 1-h followed by a 2-min warm-up and 4-min HIIT, using the “HIIT@WORK” protocol, designed by the research team of Artevelde University College Ghent (**Table 2**) and another 2 h of sitting while reading a book of their choice. In the control condition after the same 20-min breakfast, all participants simply sat for 186 min. The testing and control trials were performed in randomized order and at least 72 h apart.

**Table 2:** Order and duration of movements during the 6-min HIIT (order 1-10 “warm-up”, order 11-25 “HIIT”).

Order	Movement	Duration [s]
1	Jogging in place	10
2	Squats	15
3	Running in place	5
4	Lunge	25
5	Lunge	5
6	Squats	10
7	Squats	10
8	Running in place	5
9	Skipping in place	8
10	Jogging in place	28
11	Skipping in place	20
12	Jogging in place	10
13	Skipping in place	20
14	Jogging in place	10
15	Squats	20
16	Running in place	10
17	Squats	20
18	Jogging in place	10
19	Lunge with jumps	20
20	Jogging in place	10
21	Lunge with jumps	20
22	Jogging in place	10
23	Jumping jack	20
24	Jogging in place	10
25	Jumping Jack	20

	Sum	361


At T_0_ (after the initial 30 min of resting), T_1_ (after the 20-min breakfast), T_2_ (after sitting for 1 h), T_3_ (immediately after the 6-min HIIT), T_4_, T_5_, T_6_, and T_7_ (30, 60, 90, and 120-min after the 6-min HIIT), oxygen uptake, the respiratory exchange ratio (RER), heart rate, tissue oxygen saturation, and core and skin temperature were determined and samples of saliva and blood (from the right ear) collected. Cognitive functioning was assessed, and questionnaires answered at T_1_, T_2_, T_3_, T_4_, T_5_, T_6_, and T_7_.

### The Protocol for High-Intensity Interval Training

Each subject performed a 6-min protocol (2-min warm-up, 4-min HIIT), based on the original Tabata-protocol (Tabata et al., 1996), but using callisthenic exercises to make it more feasible and acceptable in an office-setting. The protocol involved eight forms of appropriate movement (including squats, skipping, and jumping jacks) in 10–20-s sessions at maximal intensity. During the last 4-min of the protocol, all were instructed to “do as many repetitions as you can in 20 s.” **Table 2** summarizes the order and duration of these movements during the 6-min HIIT protocol.

During all office-related testing and 6-min HIIT, the participants wore the same clothing of their own choice consisting of a pair of long trousers and a long-sleeve shirt/blouse of the sort that they would wear when performing desk work.

### Body Mass and Height

The height of the subjects standing barefoot was measured with a folding ruler. Body mass, fat and fat-free mass were assessed to the nearest 0.1 kg with a four-electrode bio-impedance scale (Model 1609N; Tanita Corporation, Tokyo, Japan) and body-mass-index (BMI; in kg m^-2^) then calculated.

### Cardio-Respiratory Measurements

During the test, heart rate, oxygen uptake, and the RER were monitored continuously with an open breath-by-breath gas analyzer (Cortex Metamax 3B, Leipzig, Germany), calibrated prior to each test with high-precision gas (15.8% O_2_, 5% O_2_ in N; Praxair, Düsseldorf, Germany) and a 3-L precision syringe. The RER was calculated by dividing the volume of carbon dioxide exhaled (VCO_2_) by the oxygen consumption (VO_2_). All gas analyses and heart rates were averaged every 30 s and these averages at T_0_, T_1_, T_2_, T_3_, T_4_, T_5_, T_6_, and T_7_ subjected to statistical analysis.

Energy expenditure (EE) during the 180 and 186 min of sitting and 6-min HIIT was calculated using the formula EE = Total energy expenditure (TEE) – resting EE (REE), where TEE = 3.9 × VO_2_ + 1.1 × VCO_2_ (Weir, 1949) and REE = 309 + 21.6 × fat-free mass (Cunningham, 1991).

### Temperature Measurement

The ambient temperature was measured with a digital thermometer (ThermoPro TP50 Digital Thermometer, itronics Inc., Toronto, ON, Canada) and aural temperature with Braun Thermoscan 7 (Kronberg, Germany). The skin temperature (T_skin_) on the hand (T_hand_), arm (T_arm_), leg (T_leg_), chest (T_chest_), back (T_back_), and thigh (T_thigh_) was obtained with a thermal imaging camera (Testo 875-11, Testo, Lenzkirch, Germany) and the mean skin temperature calculated in accordance with previous recommendations (Hardy and DuBois, 1938) as follows: T_skin_ = 0.073_hand_ + 0.163_arm_ + 0.203_chest_ + 0.193_back_ + 0.213_thigh_ +0.153_leg_. The mean body temperature (T_body_) (Ramanathan, 1964) was calculated as 0.65 T_aural_ (as a surrogate for T_core_) + 0.35 T_skin_.

### Analysis of Saliva and Blood Samples

For collection of the saliva samples as described previously (Born et al., 2016), the participants were instructed to rinse out their mouths with water, swallow any remaining saliva and then passively allow saliva to collect in the mouth while resting in a seated position with the head tilted slightly forward. After exactly 2 min, they spit the saliva accumulated into a sterile polypropylene tube (Sali-Cap Tubes, IBL International GmbH, Hamburg, Germany) through a polypropylene straw. These tubes were then stored at -80°C until analysis of cortisol (ng⋅mL^-1^) using an ELISA kit [Cortisol (Salivary) ELISA SLV-2930; DRG Instruments GmbH, Germany].

Blood samples were collected from the right earlobe in order to determine capillary blood lactate (Lactate Scout, SensLab, Germany) and glucose (Accu-Chek Aviva, Roche, Germany) concentrations with a portable device. All blood samples were analyzed in duplicate and the mean of the two measures was used for statistical analysis.

### Near-Infrared Spectroscopy

During the experiment, pre-frontal oxygenation (expressed as a tissue oxygenation index, TOI) was assessed at 6 Hz employing near-infrared spectroscopy (NIRS; NIRO 200 spectrophotometer, Hamamatsu Photonics K.K., Hamamatsu, Japan). This TOI (calculated as ([HbO_2_]+[MbO_2_])/([tHb]+[tMb]) × 100 and expressed as %) was used as an indicator of the balance between oxygen supply and consumption (Chance et al., 1992; Hamaoka et al., 1996). To standardize positioning of the NIRS as much as possible, the same researcher prepared all participants for these measurements, placing a marker on the skin to allow accurate repositioning for subsequent trials.

### Cognitive Function

All participants completed an online Stroop Color Word Test (Lenhard and Lenhard, 2011) to assess speed of information processing, executive abilities, selective attention, and the ability to inhibit habitual responses (Golden and Freshwater, 2002). Preceding this test, a standardized set of instructions explaining which keys corresponded to which colors was provided. A trial run of the Stroop test with 16 words was performed by the participants prior to the actual trial with 80 words (60 incompatible and 20 compatible). The speed and accuracy of their keyed responses following presentation of the stimulus were analyzed statistically.

### Perceptual Ratings

#### Ratings of Perceived Exertion

The participants were asked to rate their level of exertion immediately after completion of the 6-min HIIT on the Borg’s 6–20 scale (Borg, 1970).

#### Thermal Sensation

As described previously (Ha et al., 1996), thermal sensation was rated from 0 (very cold) to 9 (very hot); shivering and sweating from 1 (vigorously shivering) to 7 (heavily sweating); and clothing wetness from 1 (dry) to 4 (wet) (Brazaitis et al., 2010).

#### Profile of Mood States (POMS)

The German short version of the Profile of Mood States (Albani et al., 2005) was used to assess perceived mood (McNair et al., 1971). The 35 items (with the lead-in “I feel…?”) were rated on a 7-point scale from “not at all” (0) to “very strong” (6) and sum scores determined for anxiety/depression, fatigue, vigor and hostility.

### Statistical Analyses

All data were confirmed to be normally distributed by the Kolmogorov-Smirnov test, so that no transformation was required. The effect size, Cohen’s *d* (Cohen, 1988), was calculated for all parameters, with the thresholds for small, moderate, and large effects being set at 0.20, 0.50, and 0.80, respectively. Repeated-measures ANOVAs [time-point (T_1_–T_7_)] were executed with the different circulatory, metabolic, hormonal, thermoregulatory, cognitive, and perceptual outcome variables as the within subject factor and with SIT and SIT+HIIT as the between subject factor. A Tukey *post hoc* analysis was used where an alpha of *p* < 0.05 was considered significant. All analyses were carried out with the Statistica software package for Windows 1 (version 7.1, StatSoft Inc., Tulsa, OK, United States).

## Results

The detailed data, with statistical analysis, on each variable assessed are summarized in **Tables 3–7**.

**Table 3:** Circulatory, metabolic, and hormonal variables at the different time-points (T_0_–T_7_) while sitting (SIT) or when sitting was interrupted by the 6-min HIIT (SIT+HIIT).

	Time-points									
		
Variable	Condition	T0	T1	T2	T3	T4	T5	T6	T7	Best d
Blood glucose [mg⋅dl^-1^]	SIT	92.3 ± 7.1	112.3 ± 18.5^a^	98.9 ± 10.0	95.6 ± 11.9	100.9 ± 16.2	95.3 ± 11.6	96.6 ± 12.9	95.8 ± 10.1	0.3
	SIT+HIIT	94.2 ± 7.2	116.6 ± 17.1 ^a^	102.7 ± 15.1	93.2 ± 10.5	97.3 ± 14.3	99.3 ± 13.5	97.8 ± 15.1	92.4 ± 12.4	
Blood lactate concentration [mmol⋅l^-1^]	SIT	1.0 ± 0.2	1.2 ± 0.2	1.2 ± 0.2	1.2 ± 0.1	1.2 ± 0.1	1.2 ± 0.2	1.2 ± 0.2	1.2 ± 0.2	2.5
	SIT+HIIT	1.0 ± 0.2	1.1 ± 0.2	1.5 ± 0.4	7.6 ± 3.7^ab^	3.9 ± 3.1^ab^	2.0 ± 1.2	1.4 ± 0.4	1.2 ± 0.2	
Salivary cortisol [ng⋅mL^-1^]	SIT	11.0 ± 3.9	-	7.5 ± 3.1^a^	6.2 ± 1.8^a^	7.0 ± 3.8^a^	6.3 ± 2.4^a^	5.2 ± 1.5^a^	5.4 ± 1.5^a^	0.42
	SIT+HIIT	11.3 ± 2.0	-	7.6 ± 2.3^a^	6.8 ± 1.8^a^	8.2 ± 1.5	6.0 ± 1.2^a^	4.9 ± 1.2^a^	5.2 ± 1.1^a^	
Heart rate [bpm]	SIT	64.8 ± 9.4	72.2 ± 9.6	74.2 ± 9.8^a^	74.6 ± 8.1^a^	70.8 ± 7.9	69.6 ± 10.4	68.2 ± 10.1	69.0 ± 10.3	9.35
	SIT+HIIT	67.3 ± 8.0	71.3 ± 7.1	75.8 ± 7.7	166.0 ± 11.2^ab^	87.6 ± 10.6^ab^	82.4 ± 11.9^ab^	77.3 ± 7.4^ab^	76.5 ± 8.7^a^	
Oxygen uptake [ml⋅min^-1^]	SIT	0.240 ± 0.043	0.283 ± 0.051	0.287 ± 0.430	0.278 ± 0.042	0.269 ± 0.0067	0.247 ± 0.045	0.257 ± 0.062	0.260 ± 0.051	5.28
	SIT+HIIT	0.252 ± 0.038	0.313 ± 0.050	0.305 ± 0.057	2.673 ± 0.640^ab^	0.320 ± 0.060	0.318 ± 0.083	0.291 ± 0.056	0.306 ± 0.033	
Respiratory exchange ratio [a.u]	SIT	0.84 ± 0.05	0.84 ± 0.04	0.85 ± 0.05	0.84 ± 0.05	0.84 ± 0.05	0.85 ± 0.05	0.84 ± 0.05	0.83 ± 0.05	6.32
	SIT+HIIT	0.85 ± 0.05	0.84 ± 0.06	0.85 ± 0.06	1.30 ± 0.09^ab^	0.81 ± 0.06	0.81 ± 0.06	0.82 ± 0.06	0.80 ± 0.005	


*^*a*^*P* < 0.05 for the difference to T_*0*_ within group. ^*b*^*P* < 0.05 for the difference between groups.*

**Table 4:** Temperature (T_0_–T_7_) and ratings (T_1_–T_7_) at the different time-points while sitting (SIT) or when sitting was interrupted by the 6-min HIIT (SIT+HIIT).

	Time-points									
		
Variable	Condition	T_0_	T_1_	T_2_	T_3_	T_4_	T_5_	T_6_	T_7_	Best d
Ambient temperature [°C]	SIT	21.9 ± 0.6	22.0 ± 0.6	22.3 ± 0.4	22.4 ± 0.5	22.4 ± 0.5^a^	22.5 ± 0.4^a^	22.6 ± 0.3^a^	22.6 ± 0.3^a^	0.39
	SIT+HIIT	21.9 ± 0.7	22.2 ± 0.6	22.5 ± 0.6^a^	22.6 ± 0.6^a^	22.6 ± 0.7^a^	22.6 ± 0.7^a^	22.6 ± 0.7^a^	22.6 ± 0.7^a^	
Mean skin temperature [°C]	SIT	32.8 ± 0.6	32.7 ± 0.8	33.1 ± 0.7	33.0 ± 0.7	33.1 ± 0.9	33.1 ± 0.7	33.2 ± 0.7	33.2 ± 1.0	1.06
	SIT+HIIT	32.9 ± 0.9	32.8 ± 0.8	33.1 ± 0.9	32.2 ± 0.8^ab^	33.6 ± 1.0^a^	33.8 ± 0.9^ab^	33.5 ± 1.0^a^	33.4 ± 0.9	
Mean body temperature [°C]	SIT	35.0 ± 0.3	35.0 ± 0.3	35.1 ± 0.3	35.1 ± 0.3	35.1 ± 0.3	35.1 ± 0.4	35.2 ± 0.3	35.2 ± 0.5	0.97
	SIT+HIIT	35.1 ± 0.5	35.0 ± 0.5	35.1 ± 0.5	34.7 ± 0.5^ab^	35.3 ± 0.5	35.3 ± 0.5	35.2 ± 0.5	35.2 ± 0.5	
Thermal sensation [a.u.]	SIT	-	0.1 ± 0.7	0.1 ± 0.9	0.2 ± 0.7	0.1 ± 0.8	0.0 ± 0.9	0.2 ± 0.7	0.3 ± 0.8	2.11
	SIT+HIIT	-	0.3 ± 1.1	0.5 ± 1.0	1.9 ± 0.9^#^	0.9 ± 0.7^#^	0.5 ± 0.8	0.5 ± 0.7^#^	0.1 ± 0.8	
Sensation of sweating [a.u.]	SIT	-	0.2 ± 0.4	0.3 ± 0.5	0.3 ± 0.5	0.2 ± 0.4	0.3 ± 0.5	0.3 ± 0.5	0.2 ± 0.4	1.85
	SIT+HIIT	-	0.6 ± 1.1	0.6 ± 0.9	3.5 ± 2.4^#^	0.6 ± 0.8	0.3 ± 0.4	0.2 ± 0.3	0.3 ± 0.5	
Sensation of body odor [a.u.]	SIT	-	0.2 ± 0.3	0.2 ± 0.5	0.2 ± 0.4	0.2 ± 0.4	0.3 ± 0.4	0.2 ± 0.3	0.2 ± 0.3	1.39
	SIT+HIIT	-	0.1 ± 0.4	0.4 ± 0.8	2.4 ± 2.2^#^	1.6 ± 2.2	1.3 ± 2.1	0.9 ± 1.7	1.1 ± 1.7	


*a.u., arbitrary units. ^*a*^*P* < 0.05 for the difference to T_*0*_ for temperature and T_*1*_ for ratings within group. ^*b*^*P* < 0.05 for the difference between groups.*

**Table 5:** Cerebral oxygenation at the different time-points (T_1_–T_7_) while sitting (SIT) or when sitting was interrupted by the 6-min HIIT (SIT+HIIT).

	Time-points									
		
Variable	Condition	T_0_	T_1_	T_2_	T_3_	T_4_	T_5_	T_6_	T_7_	Best d
Left frontal lobe TSI [%]	SIT	–	100.0 ± 0.0	99.6 ± 3.8	99.3 ± 3.6	99.4 ± 4.4	99.2 ± 6.4	98.3 ± 7.8	99.1 ± 5.0	0.44
	SIT+HIIT	–	100.0 ± 0.0	99.2 ± 3.6	99.5 ± 3.1	100.4 ± 4.1	98.9 ± 3.4	97.5 ± 4.4	96.8 ± 5.5	
Right frontal lobe TSI [%]	SIT	–	100.0 ± 0.0	99.4 ± 4.5	100.0 ± 4.3	98.7 ± 5.9	99.2 ± 4.7	98.9 ± 5.8	98.5 ± 5.9	0.2
	SIT+HIIT	–	100.0 ± 0.0	98.6 ± 2.7	99.3 ± 3.5	97.9 ± 3.3	99.2 ± 3.0	99.8 ± 4.0	98.2 ± 4.3	


*^*a*^*P* < 0.05 for the difference to T_*1*_ within group. ^*b*^*P* < 0.05 for the difference between groups.*

**Table 6:** Mood states (POMS) at the different time-points (T_1_–T_7_) while sitting (SIT) or when sitting was interrupted by the 6-min HIIT (SIT+HIIT).

	Time-points									
		
Variable	Condition	T_0_	T_1_	T_2_	T_3_	T_4_	T_5_	T_6_	T_7_	Best d
Anxiety/depression [a.u.]	SIT	–	1.2 ± 2.6	0.5 ± 0.8	0.4 ± 0.8	0.4 ± 1.0	0.7 ± 1.2	0.3 ± 0.7	0.3 ± 1.2	0.69
	SIT+HIIT	–	1.7 ± 2.9^ab^	0.5 ± 0.8^a^	0.1 ± 0.3^a^	0.2 ± 0.6^a^	0.1 ± 0.3^a^	0.5 ± 0.8^a^	0.1 ± 0.3^a^	
Vigor [a.u.]	SIT	–	15.4 ± 8.3	13.4 ± 7.6	13.2 ± 8.2	12.1 ± 6.0	9.8 ± 6.7^a^	11.8 ± 8.6	12.3 ± 9.0	1.3
	SIT+HIIT	–	16.8 ± 8.8	12.9 ± 7.1	23.9 ± 8.3^ab^	18.8 ± 7.7^b^	16.1 ± 6.9^b^	15.7 ± 8.9	13.5 ± 7.9	
Fatigue [a.u.]	SIT	–	6.0 ± 4.7	5.7 ± 4.7	4.6 ± 5.0	5.0 ± 5.3	7.3 ± 7.1	6.9 ± 5.4	6.2 ± 5.3	0.49
	SIT+HIIT	–	7.5 ± 4.2	8.4 ± 6.2	3.8 ± 3.9^a^	4.8 ± 6.1	5.3 ± 5.0	6.7 ± 7.8	7.0 ± 6.6	
Hostility [a.u.]	SIT	–	0.3 ± 0.7	0.7 ± 1.2	0.5 ± 0.8	0.5 ± 1.0	1.3 ± 2.7	0.8 ± 1.3	0.3 ± 0.8	0.57
	SIT+HIIT	–	0.0 ± 0.0	0.1 ± 0.3	0.0 ± 0.0	0.0 ± 0.0	0.2 ± 0.4	0.2 ± 0.4	0.1 ± 0.3	


*a.u., arbitrary units. ^*a*^*P* < 0.05 for the difference to T_*1*_ within group. ^*b*^*P* < 0.05 for the difference between groups.*

**Table 7:** Cognitive function as assessed by the Stroop Test at the different time-points (T_1_–T_7_) during sitting (SIT) or when interrupted by the 6-min HIIT (SIT+HIIT).

	Time-points									
		
Variable	Condition	T_0_	T_1_	T_2_	T_3_	T_4_	T_5_	T_6_	T_7_	Best d
Number of correct compatible trials	SIT	–	19.4 ± 1.2	19.3 ± 0.9	19.3 ± 0.9	19.5 ± 0.7	19.3 ± 0.8	19.6 ± 0.5	19.5 ± 0.5	0.45
	SIT+HIIT	–	19.7 ± 0.7	19.5 ± 0.8	19.5 ± 1.0	19.5 ± 0.7	19.6 ± 0.5	19.6 ± 0.7	19.7 ± 0.5	
Number of incorrect compatible trials	SIT	–	0.6 ± 1.2	0.7 ± 0.9	0.7 ± 0.9	0.5 ± 0.7	0.7 ± 0.8	0.4 ± 0.5	0.6 ± 0.5	0.6
	SIT+HIIT	–	0.3 ± 0.7	0.5 ± 0.8	0.5 ± 1.0	0.5 ± 0.7	0.4 ± 0.5	0.4 ± 0.7	0.3 ± 0.5	
Number of correct incompatible trials	SIT	–	57.9 ± 1.7	57.5 ± 2.3	58.5 ± 1.4	58.2 ± 2.4	58.3 ± 2.1	58.5 ± 1.7	58.9 ± 0.9	0.37
	SIT+HIIT	–	58.1 ± 2.3	57.1 ± 2.5	57.9 ± 1.8	57.9 ± 2.3	58.2 ± 2.7	58.8 ± 1.8	58.6 ± 1.3	
Number of incorrect incompatible trials	SIT	–	2.1 ± 1.7	2.5 ± 2.3	1.5 ± 1.4	1.8 ± 2.4	1.8 ± 2.1	1.5 ± 1.7	1.1 ± 0.9	0.37
	SIT+HIIT	–	1.9 ± 2.3	2.9 ± 2.5	2.1 ± 1.8	2.1 ± 2.3	1.8 ± 2.7	1.2 ± 1.8	1.4 ± 1.3	
Duration of compatible Trials	SIT	–	628.0 ± 60.0	579.8 ± 74.7	634.0 ± 111.3	611.2 ± 87.2	583.3 ± 63.5	581.7 ± 68.9	582.4 ± 59.0	0.76
	SIT+HIIT	–	618.7 ± 69.9	606.8 ± 85.5	559.8 ± 82.2	575.6 ± 68.2	585.6 ± 96.3	604.6 ± 78.2	578.0 ± 91.3	
Duration of incompatible Trials	SIT	–	638.0 ± 80.5	638.4 ± 75.2	638.4 ± 57.9	624.0 ± 90.6	613.3 ± 78.6	601.9 ± 66.7^a^	597.3 ± 41.7	0.44
	SIT+HIIT	–	658.3 ± 70.9	630.4 ± 98.4	605.2 ± 66.0^a^	591.0 ± 53.3^a^	623.4 ± 71.2	629.1 ± 90.9^a^	622.4 ± 69.9	


*^*a*^*P* < 0.05 for the within-group difference to T_*1*_.*

Blood levels of glucose increased from T_0_ to T_1_ to the same extent in both groups. With HIIT, heart rate was higher at T_2_ and the blood level of lactate, heart rate, oxygen uptake and the RER all increased significantly at T_3_, compared to T_0_. The blood lactate concentration (at T_3_–T_4_), heart rate (at T_3_–T_6_), oxygen uptake (at T_3_) and RER were all higher with HIIT than SIT. With the exception of T_4_ following HIIT, salivary levels of cortisol declined with each successive measurement (T_1_–T_7_).

The overall energy expenditure during the 180 min of sitting and 6-min HIIT was 347 ± 47 kcal, while the corresponding value for the 186 min of sitting was 238 ± 43 kcal.

The room temperature in the office averaged 22.4 ± 0.6°C. Mean body and skin temperature were lower at T_3_ and mean skin temperature was higher at T5, and thermal sensation (at T3 + T5) and perception of sweating (at T3 and T4) higher with HIIT.

Cerebral oxygenation was similar in both groups at all time-points (**Table 5**).

The ratings of perceived exertion on the Borg’s scale after the 6-min HIIT was 14.3 ± 0.8. The POMS analysis revealed greater anxiety/depression (at T_1_) and vigor (at T_3_–T_5_) in the SIT group, with no differences in the ratings of fatigue and hostility (**Table 6**).

The numbers and duration of correct and incorrect compatible and incompatible trials during the Stroop test were also the same in both groups (**Table 7**).

## Discussion

Although several studies have focused on the psycho-physiological effects of interrupting prolonged sitting with light-to-moderate activity, to the best of our knowledge this is the first to investigate various psycho-physiological responses to the interruption of sitting by a 6-min micro-session of HIIT.

Overall, interruption of prolonged sitting with a 6-min session of HIIT induced more pronounced circulatory and metabolic responses and improved certain aspects of perception, without affecting the hormonal, thermoregulatory or cognitive functions examined.

### Acute Circulatory, Metabolic and Hormonal Responses

The 6-min HIIT induced a 2.3-fold increase in heart rate, from approximately 70–75 beats⋅min^-1^ at T_1_ and T_2_ to 166 ± 11 beats⋅min^-1^ at T_3_ (i.e., immediately after the 6-min HIIT), the latter corresponding to 85 ± 6% of peak heart rate during maximal ramp cycling. Despite the higher peak heart rate during ramp treadmill running than ramp cycling, the increase in HR from T_2_ to T_3_ was clearly higher than the difference reported previously between walking for 30 min and then sitting or between sitting with or without 1 min 40 s of walking every 30 min (approximately 85 beats⋅min^-1^) (Peddie et al., 2013). In the present investigation peak oxygen uptake (ml min^-1^ kg^-1^) reached 84 ± 7% of the maximal value, which, as confirmed by the “strenuous” ratings of perceived exertion (14.3 ± 0.8 on the Borg’s scale), may be classified as vigorous exercise. In addition, with the 6-min HIIT the level of blood lactate rose to 7.6 ± 3.7 mmol L^-1^, indicating involvement of considerable anaerobic metabolism.

Numerous studies have verified that accelerated metabolism (as reflected in excess post-oxygen consumption, EPOC) is important in connection with weight control (Gore and Withers, 1990; Brockman et al., 1993; Quinn et al., 1994; Laforgia et al., 1997; Hunter et al., 1998). In the present case, the overall energy expenditure during the 3 h of sitting and 6-min HIIT was approximately 347 ± 47 kcal, whereas the corresponding value for the 186 min of sitting was 238 ± 43 kcal. From a practical perspective, such a difference, if attained daily, would lead to usage of an additional 765 kcal of EE per week. Thus, when performed more than once each day (e.g., every other hour), this type of active break could help maintain and/or lose weight, as well as reduce the time spent sedentary.

In contexts like ours, the RER has been utilized to quantify substrate utilization. Reduced RER is indicative of enhanced lipid oxidation and down-regulation of glycolysis (Dudley et al., 1987; Jansson and Kaijser, 1987; Brooks, 1997; Bergman and Brooks, 1999). Here, the RER when sitting after the 6-min HIIT was lower than when sitting without exercise. This finding is consistent with the previous observation that regularly interrupting sitting with brief sessions of physical activity (twenty 90-s bouts over a period of 8 h) potently reduces postprandial serum levels of triglycerides in postmenopausal women (Miyashita et al., 2016).

In the current investigation the postprandial capillary level of glucose while sitting after the 6-min HIIT remained within the normal range for our students. Similarly, with normal-weight adults interruption of 2.5 h of sitting with 2 min of walking every 20 min did not alter postprandial blood levels of glucose (Hansen et al., 2016). Conversely, in healthy young adults hourly interruption of prolonged (8 h) sitting with physical activity (i.e., 8 min of moderate-intensity cycling involving muscle activity 7–8-fold the resting value) attenuates postprandial levels of C-peptide, but does not alter other cardio-metabolic biomarkers (Altenburg et al., 2013).

### Hormonal Responses

Another major finding here is the lack of any difference in salivary levels of cortisol between the SIT and SIT+HIIT groups. Cortisol plays a central role in physiological and behavioral responses to challenges that activate the hypothalamic–pituitary–adrenal axis to stimulate release of this hormone from the adrenal cortex (Tsai et al., 2010). Salivary levels of cortisol reflect psycho-physiological stress during both single and repeated bouts of exercise (Filaire et al., 2001; Thatcher et al., 2004), as well as circulating levels of free cortisol, which are recommended for use as an indicator of training stress (Passelergue and Lac, 1999). Accordingly, we conclude that the 6-min HIIT exerted no pronounced effect on the level of stress, at least based on analysis of salivary cortisol.

### Thermoregulation Following the 6-Min HIIT

Recovery from a session of exercise is associated with elevated metabolism, which reflects the effect of the exercise on the individual’s temperature (LaForgia et al., 2006). The skin and body temperature at T_3_ (i.e., after termination of the 6-min HIIT) were slightly lower, potentially due to the flow of air generated by the movement. The sensations of heat and body odor were enhanced after the HIIT and certain individuals may perceive this as less desirable, which might make them disinclined to participate frequently in this type of exercise.

In addition, from a practical point of view, it is noteworthy that our subject’s heart rates had returned to baseline approximately 60 min after the 6-min of HIIT. It is well known that normalization of the circulation following high-intensity exercise is due to beta-adrenergic stimulation (LaForgia et al., 2006), as well as that circulatory responses are stimulated in order to disseminate heat. Based on our present findings, our advice is that if this 6-min HIIT is performed more often than once, at least 60 min of recovery should be allowed between bouts in order for circulation to return to normal.

### Cognitive Function, Cerebral Oxygenation, and Mood

Meta-analyses indicate acute exercise exerts beneficial effects on cognition in the general population. Enhanced metabolic activity in the brain in connection with acute bouts of submaximal (Endo et al., 2013; Giles et al., 2014) and high-intensity (Bediz et al., 2016) exercise is associated with improved cerebral oxygenation and performance of cognitive tasks. Neural consumption of energy requires elevated cerebral blood flow to meet oxygen and glucose demands. For this reason, we employed cerebral oxygenation (assessed by near-infrared spectroscopy) as an indicator of changes in cerebral hemodynamics and cognitive workload (evaluated by the Stroop task). Neither of these variables differed between the groups. Interestingly HIIT+SIT induced a feeling of greater vigor, which might help motivate the desk worker.

### Limitations of the Present Study

Our protocol did not involve comparison to low-intensity exercise, which could have provided a more definitive conclusion concerning whether high- or low-intensity exercise in connection with desk work is more beneficial with respect to circulatory, metabolic, hormonal, thermal, cognitive, and perceptual responses. In addition, cognitive functioning during sitting for longer than 3 h might have been affected by HIIT. Moreover, we included only a single 6-min HIIT break and a larger number of such breaks might have resulted in different conclusions concerning the SIT and SIT+HIIT. Although, a rectal measurement is the golden standard for determination of core temperature, we refrained for employing this methodology because of the prolonged sitting involved.

## Conclusion

Overall, interruption of prolonged sitting by a 6-min HIIT induced more pronounced circulatory and metabolic responses and improved certain aspects of perception (i.e., vigor), without affecting hormonal, thermoregulatory or cognitive functions. The overall energy expenditure during the 3 h of sitting and 6-min HIIT was approximately 347 ± 47 kcal, whereas the corresponding value for the 186 min of sitting was 238 ± 43 kcal. From a practical perspective, such a difference, if attained daily, would lead to usage of an additional 765 kcal of EE per week. Thus, when performed more than once each day (e.g., every other hour), this type of active break could assist in body mass management as well as reduce the time spent sedentary.

## Author Contributions

All authors have made a substantial, direct, and intellectual contribution to the work and approved it for publication.

## Conflict of Interest Statement

The authors declare that the research was conducted in the absence of any commercial or financial relationships that could be construed as a potential conflict of interest.
